# Point-to-Point Macular Structure–Function Relationships in Healthy and Glaucomatous Eyes Using OCT and Microperimetry

**DOI:** 10.3390/jcm15093312

**Published:** 2026-04-27

**Authors:** Jose Javier Garcia-Medina, Paloma Sobrado-Calvo, Lorena Lopez-Canovas, Maria Dolores Lopez-Bernal, Maria Dolores Pinazo-Duran, Vicente Zanon-Moreno, Monica Del-Rio-Vellosillo

**Affiliations:** 1 General University Hospital Morales Meseguer, 30008 Murcia, Spainmonicadelriov@hotmail.com (M.D.-R.-V.); 2 Department of Ophthalmology, Optometry, Otolaryngology and Pathology, University of Murcia, 30100 Murcia, Spain; sobrado@um.es (P.S.-C.); lorena.l.c@um.es (L.L.-C.); 3 Ophthalmic Research Unit “Santiago Grisolia”/FISABIO, 46017 Valencia, Spain; vicente.zanon-moreno@uv.es; 4 Cooperative Research Network Oriented to Results in Health on Inflammatory Diseases, Institute of Health Carlos III (RICORS-REI, RD24/0002/0007), 28029 Madrid, Spain; 5 Multidisciplinary Research Group in Vision and Ophthalmology, University of Murcia, 30003 Murcia, Spain; 6 Cellular and Molecular Ophthalmobiology Group, Surgery Department, Faculty of Medicine and Dentistry, University of Valencia, 46010 Valencia, Spain; 7 Department of Preventive Medicine and Public Health, Faculty of Medicine and Dentistry, University of Valencia, 46010 Valencia, Spain

**Keywords:** glaucoma, optical coherence tomography, microperimetry, structure–function relationship, ganglion cell complex, macular sensitivity, retinal layer thickness, point-to-point analysis

## Abstract

**Purpose**: To explore anatomically adjusted point-to-point relationships between macular sensitivity and intraretinal layer thickness in healthy and glaucomatous eyes using combined optical coherence tomography (OCT) and microperimetry. **Methods**: Seventy-two eyes were included (27 healthy controls and 45 eyes with primary open-angle glaucoma). Retinal sensitivity was assessed using MP-1 microperimetry, and retinal structure was evaluated with Spectralis OCT. Automatic segmentations included macular retinal nerve fiber layer (mRNFL), ganglion cell layer (GCL), inner plexiform layer (IPL), GCL + IPL, ganglion cell complex (mRNFL + GCL + IPL), and the outer retinal layer. Microperimetry maps were anatomically aligned with OCT grids using vascular landmarks, and ganglion cell displacement was considered when analyzing inner retinal layers. Thickness measurements were obtained at corresponding anatomical points, and structure–function associations were assessed using Spearman correlation analysis to generate spatial correlation maps. **Results**: Almost no significant pointwise correlations were detected in healthy eyes across any retinal segmentation. In glaucomatous eyes, significant positive correlations were observed for inner retinal layers, whereas no significant associations were found for the outer retinal layer. Distinct spatial patterns were identified, with peripheral correlations for mRNFL and paracentral temporal correlations for GCL, IPL, and GCL + IPL. The highest number of significant associations was observed for the ganglion cell complex. **Conclusions**: Anatomically adjusted pointwise analysis revealed localized and heterogeneous patterns of macular structure–function coupling predominantly involving ganglion cell-related layers in glaucoma. High-resolution mapping may uncover spatial relationships that are partially obscured by regional or spatially averaged approaches and should be interpreted as a complementary exploratory strategy rather than a replacement for established regional analyses.

## 1. Introduction

Glaucoma is a leading cause of irreversible blindness worldwide and is characterized by progressive loss of retinal ganglion cells and their axons, resulting in structural and functional impairment of the retina. In addition to ganglion cell degeneration, multiple intraretinal sublayers may be affected, reflecting the complex neurodegenerative processes underlying glaucomatous damage. Early detection and monitoring of structural and functional alterations are essential to prevent irreversible visual loss. Optical coherence tomography (OCT) and microperimetry provide complementary structural and functional information, enabling detailed assessment of the macula.

Microperimetry evaluates retinal sensitivity by projecting stimuli onto predefined retinal locations while tracking eye movements, allowing precise mapping of functional deficits at specific anatomical sites [1,2]. Optical coherence tomography is a non-invasive imaging modality that provides high-resolution cross-sectional images of retinal layers, enabling quantitative analysis of intraretinal segmentations such as the retinal nerve fiber layer and ganglion cell–inner plexiform layer complex, which are notably affected in glaucoma [3,4]. Although responses reflect the integrity of the entire visual pathway, the term ‘macular sensitivity’ is used to denote functional responses elicited by stimuli projected onto anatomically defined macular regions.

Numerous studies have demonstrated significant associations between functional impairment and structural thinning of inner retinal layers in glaucomatous eyes, contributing to the understanding of disease mechanisms and potential biomarkers for progression [5,6,7,8]. Most investigations have relied on regional or averaged analyses, examining correlations across relatively large retinal areas. Although these approaches are clinically robust, spatial averaging may partially obscure localized structure–function relationships within the macula.

Point-to-point analysis has been proposed as a strategy to explore structure–function coupling at a finer spatial scale [9,10]. However, recent work by Giammaria et al. suggested that increasing spatial precision does not necessarily result in stronger correlations, emphasizing the influence of spatial summation, measurement variability, and patterns of structural damage in macular analyses [11]. These findings highlight the need to interpret high-resolution mapping primarily within an exploratory and mechanistic framework rather than as a replacement for clinically established regional analyses.

Different functional testing strategies may also influence the interpretation of structure–function relationships. Fundus-tracking perimetry systems, such as the Compass perimeter, aim to improve psychophysical robustness and reproducibility of visual field measurements, whereas microperimetry provides direct anatomical localization of retinal stimuli. While enhanced threshold stability may improve functional consistency, anatomically localized testing may be advantageous when investigating spatially refined structure–function coupling.

In previous work, our group reported regional macular structure–function relationships using superpixel-based analysis of the posterior pole OCT grid [12]. Those studies demonstrated physiologically meaningful regional associations but did not evaluate anatomically adjusted point-to-point correlations accounting for ganglion cell displacement. Therefore, the aim of the present study was to explore anatomically adjusted point-to-point structure–function relationships between retinal sensitivity and intraretinal layer thickness in healthy and glaucomatous eyes using combined OCT and microperimetry mapping.

## 2. Materials and Methods

### 2.1. Study Design and Participants

This observational cross-sectional study represents an anatomically adjusted secondary analysis derived from a previously characterized cohort of healthy subjects and patients with primary open-angle glaucoma exhibiting treat to fixation, indicating functional involvement of the central visual field. Inclusion and exclusion criteria are defined elsewhere [12]. This inclusion criterion ensures macular involvement and allows a focused analysis of central structure–function relationships. This selection specifically targets patients with macular involvement rather than peripheral-dominant early disease, thereby allowing a focused evaluation of central structure–function relationships.

Written informed consent was obtained from all participants.

### 2.2. Microperimetry

**Figure 1:**
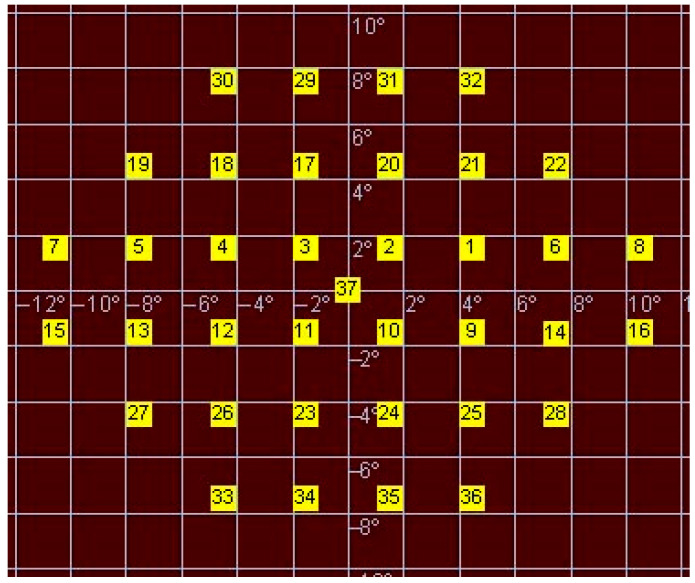
Microperimetry examination pattern. Pattern of the 36 microperimetry examinations (location number 37 was not considered for the analysis) used for anatomically adjusted point-to-point analysis. Retinal sensitivity was assessed using a 4–2–1 threshold strategy with a Goldmann III stimulus (200 ms duration). Sensitivity values were recorded in decibels (dB) under mesopic background luminance (4 apostilbs), with a maximum stimulus luminance of 20 dB. The spatial arrangement of test locations defined the functional framework for subsequent structure–function correspondence.

Macular retinal sensitivity was assessed using the MP-1 microperimeter (Nidek Technologies, Padova, Italy). A predefined posterior pole stimulus pattern was used with a Goldmann III stimulus size, 200 ms duration, and a 4–2–1 threshold strategy under mesopic background luminance (4 apostilbs), with a maximum stimulus luminance of 20 dB. Sensitivity values were recorded in decibels (dB) and used for point-to-point structure–function analysis at 36 locations (Figure 1).

### 2.3. Optical Coherence Tomography

Structural imaging was performed using spectral-domain OCT (Spectralis, Heidelberg Engineering, Heidelberg, Germany) with the posterior pole 8 × 8 grid protocol. Automated segmentation provided thickness measurements for the macular retinal nerve fiber layer (mRNFL), ganglion cell layer (GCL), inner plexiform layer (IPL), GCL + IPL, ganglion cell complex (mRNFL + GCL + IPL), and outer retinal layer.

### 2.4. Anatomical Overlay and Point-to-Point Thickness Extraction

**Figure 2:**
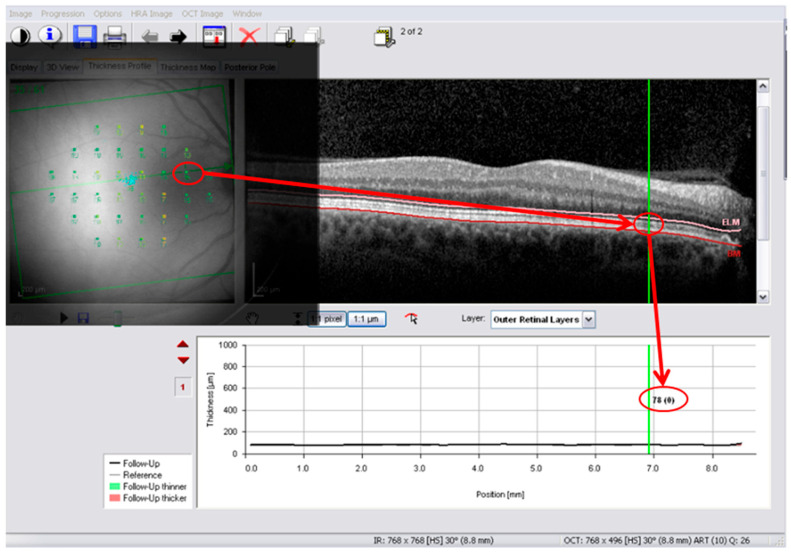
Anatomical overlay for outer retinal layer thickness extraction. OCT interface with floating microperimetry overlay aligned using anatomical references. After fixation of the floating window, the operator interacted directly with the OCT software (6.0 software version) to obtain thickness values at each microperimetry location. Thickness estimation was obtained by pointing at each test location on the fundoscopic image and retrieving the corresponding structural value on the posterior pole map. In this example, outer retinal layer segmentation is illustrated; no ganglion cell displacement correction was applied.

Microperimetry maps were anatomically superimposed onto OCT posterior pole images using vascular landmarks and optic nerve head references through Overlay2 software (version 2.0, available at https://overlaysoftware.com). A floating microperimetry window was fixed over the OCT interface, allowing direct visualization of functional test locations relative to structural maps. Thickness values corresponding to each microperimetry target were obtained by pointing to the anatomically matched location on the OCT posterior pole map (Figure 2). Eyes in which reliable anatomical correspondence between microperimetry and OCT posterior pole maps could not be achieved were excluded prior to analysis. Overlay2 operates by generating a semi-transparent floating image that is fixed relative to the OCT interface, enabling simultaneous visualization and interaction with the underlying structural image.

**Figure 3:**
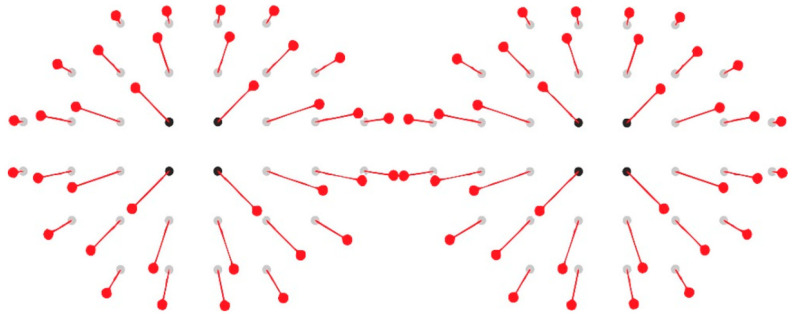
Ganglion cell displacement pattern for anatomically adjusted measurements. Schematic illustration of the application of a ganglion cell displacement model for anatomically adjusted structure–function analysis, based on previously described methods [13]. Gray reference points represent original microperimetry test locations, whereas displaced red points indicate anatomically corrected positions used for thickness extraction in ganglion cell–related segmentations (mRNFL, GCL, and IPL). The illustration is intended for conceptual visualization purposes.

For inner retinal segmentations, ganglion cell displacement was considered to improve anatomical correspondence between functional stimulation sites and structural measurements. A displacement model adapted from Turpin et al. [13] was superimposed onto the microperimetry grid, generating anatomically corrected measurement points (Figure 3).

**Figure 4:**
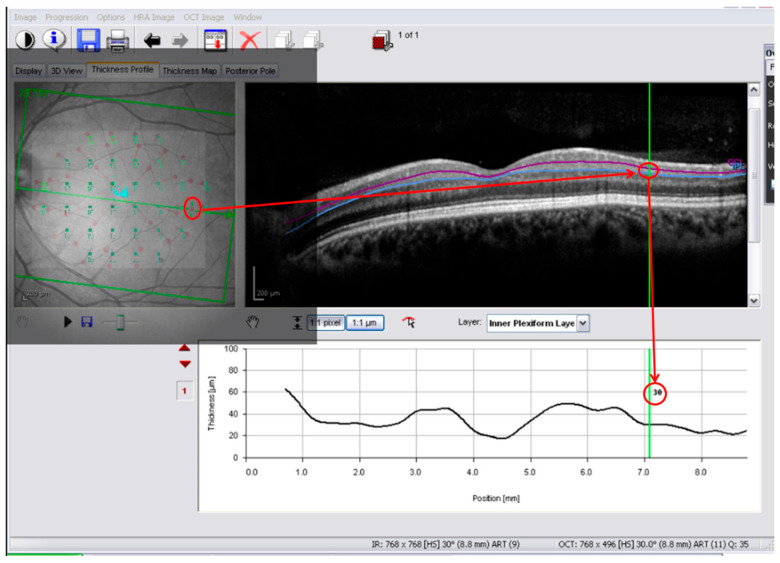
Anatomically adjusted overlay for inner retinal layer thickness extraction. OCT interface with floating microperimetry overlay including the ganglion cell displacement pattern. After anatomical alignment, thickness values were obtained by pointing at displaced microperimetry locations, allowing anatomically corrected extraction of intraretinal thickness. The example illustrates inner plexiform layer (IPL) segmentation.

Thickness extraction at displaced locations was performed using the same floating overlay technique (Figure 4). The thickness extraction for all the individual segmentations was performed by the same experienced operator (L.L.-C.). The thickness of the GCL + IPL (combined inner layers) and the ganglion cell complex (mRNFL + GCL + IPL) was calculated by summing the thicknesses of the individual layers.

### 2.5. Statistical Analysis

Data were analyzed using SPSS (v22.0, SPSS Inc., Chicago, IL, USA). Point-to-point structure–function relationships were assessed using Spearman rank correlation coefficients between retinal sensitivity and intraretinal thickness values at corresponding anatomical locations. Correlation maps were generated for each retinal layer, and statistical significance was defined as *p* < 0.05. Correlation strength was interpreted as follows: <0.20 very weak, 0.20–0.40 weak, 0.40–0.60 moderate, 0.60–0.80 strong, and >0.80 very strong.

## 3. Results

### 3.1. Study Population

Seventy-two eyes were included in the final anatomically adjusted point-to-point analysis, comprising 27 healthy control eyes and 45 eyes with primary open-angle glaucoma (POAG). Eyes excluded from the original cohort did not differ clinically but were removed due to insufficient anatomical correspondence between microperimetry and OCT posterior pole maps. Demographic and clinical characteristics of the included population are summarized in Table 1.

### 3.2. Layer-Specific Point-to-Point Structure–Function Relationships

#### 3.2.1. Outer Retinal Layer

**Figure 5:**
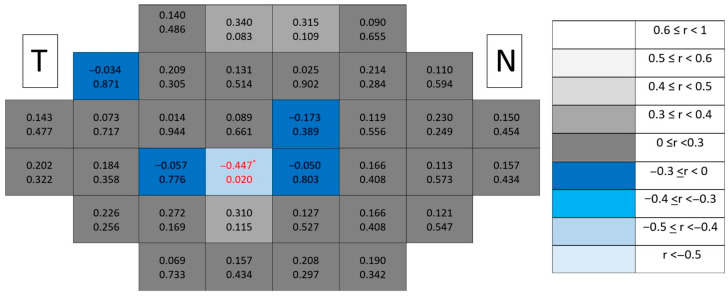
Point-to-point correlations between retinal sensitivity and outer retinal layer thickness in healthy controls. Each cell represents the correlation between point-to-point thickness of the considered segmentation and sensitivity. The number on the top indicates the Spearman rho coefficient and the number on the bottom the significance (*p*). If *p* < 0.05, the numbers of the cell are indicated in red in order to highlight significant results. The degree of correlation is represented according to the scale on the right. The schemes depict the results as if all eyes were right eyes. T and N indicate Temporal and Nasal regions, respectively. * *p* < 0.05.

Structure–function coupling at the outer retinal level remained negligible in healthy controls (Figure 5). Correlation coefficients were uniformly low across the posterior pole, and only a single isolated significant location (*p* < 0.05) was detected. This isolated finding lacked spatial continuity and did not follow any recognizable anatomical gradient.

**Figure 6:**
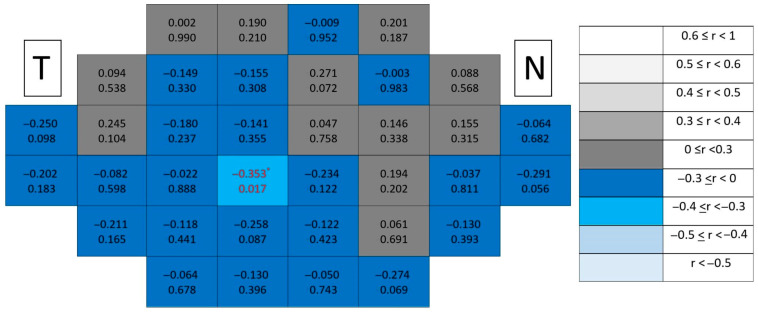
Point-to-point correlations between retinal sensitivity and outer retinal layer thickness in glaucomatous eyes. Each cell represents the correlation between point-to-point thickness of the considered segmentation and sensitivity. The number on the top indicates the Spearman rho coefficient and the number on the bottom the significance (*p*). If *p* < 0.05, the numbers of the cell are indicated in red in order to highlight significant results. The degree of correlation is represented according to the scale on the right. The schemes depict the results as if all eyes were right eyes. T and N indicate Temporal and Nasal regions, respectively. * *p* < 0.05.

Structure–function coupling at the outer retinal level remained negligible in glaucomatous eyes (Figure 6), with only a single isolated significant location and absence of spatial continuity across the posterior pole. Compared with inner retinal layers, the outer retina consistently exhibited the lowest number of significant associations and the weakest correlation magnitudes.

#### 3.2.2. Macular Retinal Nerve Fiber Layer (mRNFL)

**Figure 7:**
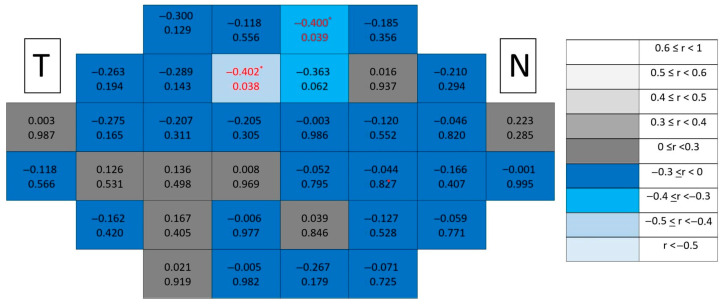
Point-to-point correlations between retinal sensitivity and mRNFL thickness in healthy controls. Each cell represents the correlation between point-to-point thickness of the considered segmentation and sensitivity. The number on the top indicates the Spearman rho coefficient and the number on the bottom the significance (*p*). If *p* < 0.05, the numbers of the cell are indicated in red in order to highlight significant results. The degree of correlation is represented according to the scale on the right. The schemes depict the results as if all eyes were right eyes. T and N indicate Temporal and Nasal regions, respectively. * *p* < 0.05.

Correlation maps in healthy controls (Figure 7) revealed diffuse low-level associations across the posterior pole. Significant cells appeared sporadically and did not form contiguous clusters, reflecting the absence of spatially coherent structure–function coupling under physiological conditions.

**Figure 8:**
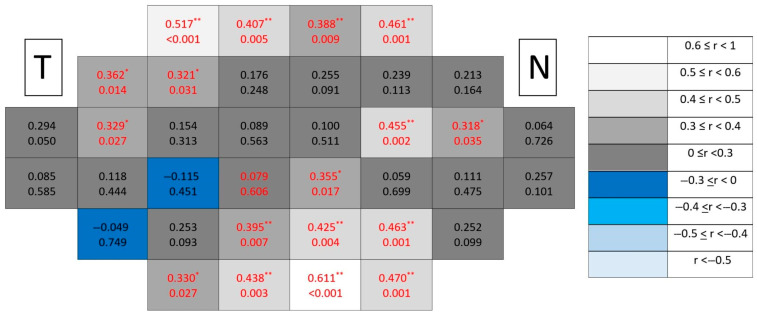
Point-to-point correlations between retinal sensitivity and mRNFL thickness in glaucomatous eyes. Each cell represents the correlation between point-to-point thickness of the considered segmentation and sensitivity. The number on the top indicates the Spearman rho coefficient and the number on the bottom the significance (*p*). If *p* < 0.05, the numbers of the cell are indicated in red in order to highlight significant results. The degree of correlation is represented according to the scale on the right. The schemes depict the results as if all eyes were right eyes. T and N indicate Temporal and Nasal regions, respectively. * *p* < 0.05 and ** *p* < 0.01.

In glaucomatous eyes (Figure 8), correlations exhibited greater spatial organization. Significant associations preferentially emerged in peripheral macular regions, particularly along superior and inferior sectors, forming localized clusters. Despite this increase in spatial structure compared with controls, both the number and magnitude of correlations remained lower than those observed in combined ganglion cell-related segmentations.

#### 3.2.3. Ganglion Cell Layer (GCL)

**Figure 9:**
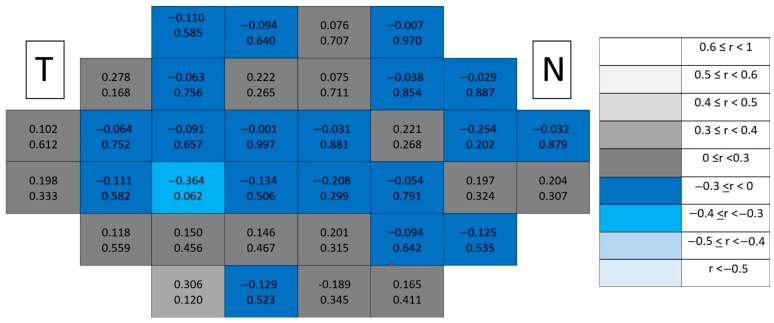
Point-to-point correlations between retinal sensitivity and GCL thickness in healthy controls. Each cell represents the correlation between point-to-point thickness of the considered segmentation and sensitivity. The number on the top indicates the Spearman rho coefficient and the number on the bottom the significance (*p*). If *p* < 0.05, the numbers of the cell are indicated in red in order to highlight significant results. The degree of correlation is represented according to the scale on the right. The schemes depict the results as if all eyes were right eyes. T and N indicate Temporal and Nasal regions, respectively.

Healthy control maps (Figure 9) demonstrated no correlations and absence of clustering. 

**Figure 10:**
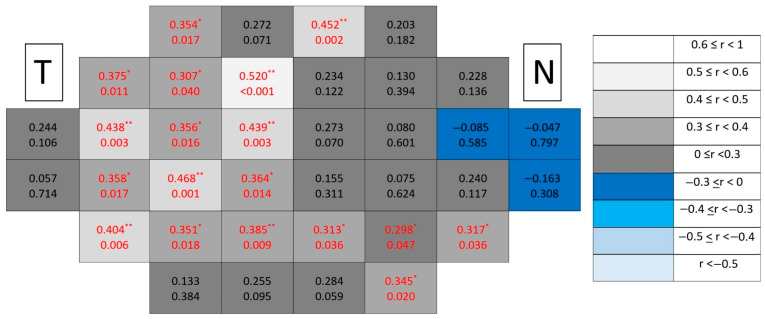
Point-to-point correlations between retinal sensitivity and GCL thickness in glaucomatous eyes. Each cell represents the correlation between point-to-point thickness of the considered segmentation and sensitivity. The number on the top indicates the Spearman rho coefficient and the number on the bottom the significance (*p*). If *p* < 0.05, the numbers of the cell are indicated in red in order to highlight significant results. The degree of correlation is represented according to the scale on the right. The schemes depict the results as if all eyes were right eyes. T and N indicate Temporal and Nasal regions, respectively. * *p* < 0.05 and ** *p* < 0.01.

In contrast, in glaucomatous eyes (Figure 10), significant correlations showed a distinctive paracentral temporal distribution, forming localized clusters adjacent to the fovea. Compared with mRNFL maps, GCL correlations displayed greater spatial compactness and higher rho values, indicating stronger localized associations.

#### 3.2.4. Inner Plexiform Layer (IPL)

**Figure 11:**
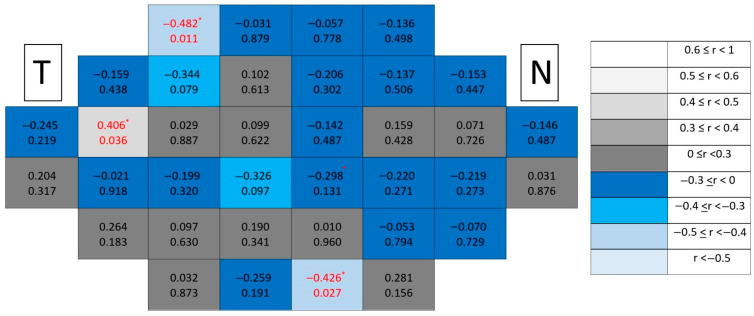
Point-to-point correlations between retinal sensitivity and IPL thickness in healthy controls. Each cell represents the correlation between point-to-point thickness of the considered segmentation and sensitivity. The number on the top indicates the Spearman rho coefficient and the number on the bottom the significance (*p*). If *p* < 0.05, the numbers of the cell are indicated in red in order to highlight significant results. The degree of correlation is represented according to the scale on the right. The schemes depict the results as if all eyes were right eyes. T and N indicate Temporal and Nasal regions, respectively. * *p* < 0.05.

Correlation maps in healthy eyes (Figure 11) remained spatially diffuse with predominantly low coefficients and absence of clustering.

**Figure 12:**
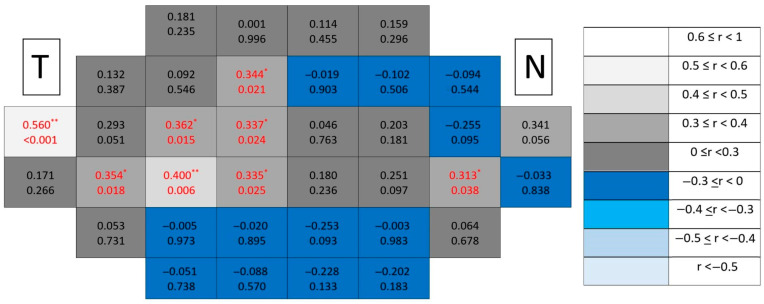
Point-to-point correlations between retinal sensitivity and IPL thickness in glaucomatous eyes. Each cell represents the correlation between point-to-point thickness of the considered segmentation and sensitivity. The number on the top indicates the Spearman rho coefficient and the number on the bottom the significance (*p*). If *p* < 0.05, the numbers of the cell are indicated in red in order to highlight significant results. The degree of correlation is represented according to the scale on the right. The schemes depict the results as if all eyes were right eyes. T and N indicate Temporal and Nasal regions, respectively. * *p* < 0.05 and ** *p* < 0.01.

In glaucomatous eyes (Figure 12), significant correlations again demonstrated a paracentral temporal predominance, forming compact clusters partially overlapping with those observed for the GCL.

#### 3.2.5. GCL + IPL (Combined Inner Layers)

**Figure 13:**
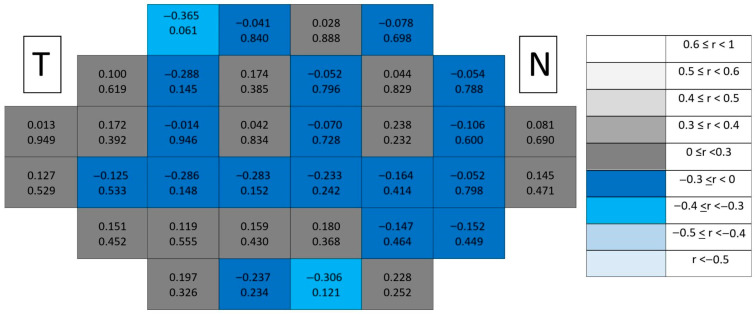
Point-to-point correlations between retinal sensitivity and GCL + IPL thickness in healthy controls. Each cell represents the correlation between point-to-point thickness of the considered segmentation and sensitivity. The number on the top indicates the Spearman rho coefficient and the number on the bottom the significance (*p*). If *p* < 0.05, the numbers of the cell are indicated in red in order to highlight significant results. The degree of correlation is represented according to the scale on the right. The schemes depict the results as if all eyes were right eyes. T and N indicate Temporal and Nasal regions, respectively.

Healthy control maps (Figure 13) maintained no correlations and spatially homogeneous patterns without clustering.

**Figure 14:**
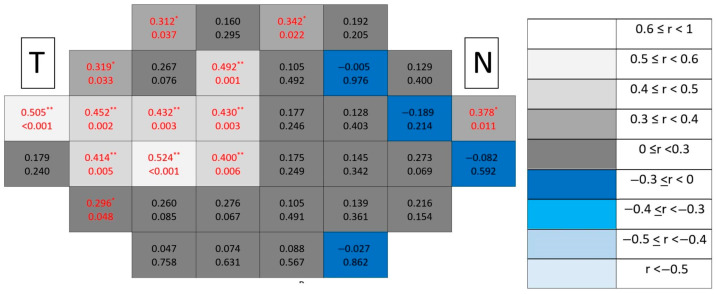
Point-to-point correlations between retinal sensitivity and GCL + IPL thickness in glaucomatous eyes. Each cell represents the correlation between point-to-point thickness of the considered segmentation and sensitivity. The number on the top indicates the Spearman rho coefficient and the number on the bottom the significance (*p*). If *p* < 0.05, the numbers of the cell are indicated in red in order to highlight significant results. The degree of correlation is represented according to the scale on the right. The schemes depict the results as if all eyes were right eyes. T and N indicate Temporal and Nasal regions, respectively. * *p* < 0.05 and ** *p* < 0.01.

However, in glaucomatous eyes (Figure 14), significant correlations formed more coherent paracentral temporal clusters than in individual layers, suggesting increased spatial coherence when combining synaptic and ganglion cell components.

#### 3.2.6. Ganglion Cell Complex (mRNFL + GCL + IPL)

**Figure 15:**
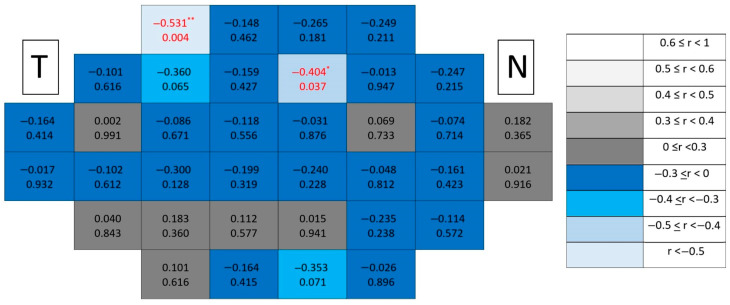
Point-to-point correlations between retinal sensitivity and ganglion cell complex thickness in healthy controls. Each cell represents the correlation between point-to-point thickness of the considered segmentation and sensitivity. The number on the top indicates the Spearman rho coefficient and the number on the bottom the significance (*p*). If *p* < 0.05, the numbers of the cell are indicated in red in order to highlight significant results. The degree of correlation is represented according to the scale on the right. The schemes depict the results as if all eyes were right eyes. T and N indicate Temporal and Nasal regions, respectively. * *p* < 0.05 and ** *p* < 0.01.

Healthy control maps (Figure 15) exhibited uniformly low correlations across the posterior pole without spatial organization.

**Figure 16:**
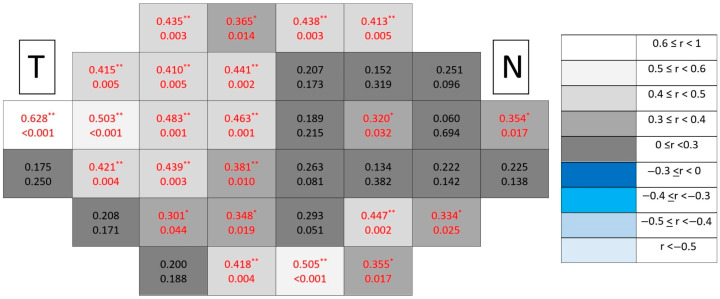
Point-to-point correlations between retinal sensitivity and ganglion cell complex thickness in glaucomatous eyes. Each cell represents the correlation between point-to-point thickness of the considered segmentation and sensitivity. The number on the top indicates the Spearman rho coefficient and the number on the bottom the significance (*p*). If *p* < 0.05, the numbers of the cell are indicated in red in order to highlight significant results. The degree of correlation is represented according to the scale on the right. The schemes depict the results as if all eyes were right eyes. T and N indicate Temporal and Nasal regions, respectively. * *p* < 0.05 and ** *p* < 0.01.

Among all analyzed segmentations, the ganglion cell complex emerged as the dominant spatial substrate of structure–function coupling in glaucomatous eyes (Figure 16), concentrating both the highest number of significant locations and the strongest correlation coefficients. Clusters formed broader contiguous regions combining peripheral and paracentral temporal areas, clearly exceeding the spatial extent observed in individual inner retinal layers.

## 4. Discussion

The present study explored anatomically adjusted point-to-point macular structure–function relationships and demonstrated that localized correlations are predominantly confined to ganglion cell-related layers, with modest strength and heterogeneous spatial distribution. Significant associations were absent in healthy eyes and largely restricted to inner retinal segmentations in glaucomatous eyes, with the ganglion cell complex concentrating the highest number and intensity of correlations. In contrast, outer retinal layers exhibited minimal or absent pointwise associations. These findings suggest that increasing analytical resolution exposes intrinsic biological variability rather than consistently reinforcing structure–function coupling. Clinically, these findings support the use of high-resolution mapping as a complementary tool to better characterize spatial heterogeneity in macular damage. This observation may be explained by the lack of direct functional contribution of outer retinal layers to psychophysical sensitivity in glaucoma, as well as increased noise at pointwise resolution compared to regional averaging. Point-to-point mapping may be particularly useful in detecting localized macular damage and spatial heterogeneity that could be overlooked by global ganglion cell complex metrics.

Structure–function relationships in glaucoma have traditionally been interpreted using regional analytical frameworks, where spatial averaging enhances statistical robustness and reflects the integrative behavior of retinal ganglion cell populations [5,6]. Early investigations demonstrated that correlations between structural measures and visual sensitivity are constrained by neural pooling, receptive field overlap, and non-linear relationships between anatomical damage and functional loss [8]. Subsequent macular OCT studies confirmed that ganglion cell-related layers provide the strongest structural correlates of central visual function [7], while localized analyses attempted to refine this relationship through spatially constrained approaches [9,10]. However, increasing spatial resolution frequently reveals greater heterogeneity rather than stronger associations.

From a physiological perspective, functional sensitivity reflects the integrated output of retinal circuitry rather than a strict one-to-one correspondence with local anatomical measurements. Receptive field organization and ganglion cell density gradients introduce spatial summation phenomena that inherently limit pointwise correlations [14,15]. These mechanisms likely explain why combined ganglion cell complex measurements exhibited stronger associations than individual layers, as integrated structural metrics more accurately approximate the neural substrate underlying visual sensitivity [16,17]. The predominance of paracentral temporal correlations observed in glaucomatous eyes is consistent with known patterns of macular vulnerability and asymmetric ganglion cell distribution.

Contemporary investigations comparing functional testing strategies further support the notion that increasing anatomical precision does not necessarily strengthen structure–function concordance. Studies comparing standard automated perimetry with microperimetry reported comparable macular correlations despite differences in retinal localization [18], and fundus-tracking perimetry improved fixation stability without markedly increasing correlation strength [19]. Within this framework, the work by Giammaria et al. [11] provides a particularly relevant reference point. Using retina-referenced perimetry, the authors demonstrated that localized macular analysis yields heterogeneous and modest correlations, emphasizing that enhanced spatial precision primarily reveals intrinsic variability rather than stronger coupling. Although methodological differences exist between both studies, the two approaches converge conceptually, suggesting that high-resolution spatial mapping may be more informative for mechanistic exploration than for strengthening clinical structure–function metrics.

The absence of consistent outer retinal correlations observed in the present study contrasts with findings from regional analyses, including our previous work [12]. This discrepancy is physiologically plausible, as glaucoma primarily affects inner retinal structures. When spatial averaging is minimized, co-localization effects between layers are reduced, revealing the relative independence of outer retinal thickness from functional impairment. Clinically, the disappearance of outer retinal correlations at the pointwise level reinforces the concept that functional loss in glaucoma is primarily driven by ganglion cell pathology and that outer retinal associations observed in regional analyses may reflect structural covariance rather than direct functional coupling.

Additional insight arises from considering anatomical displacement and non-linear structure–function dynamics. The displacement of ganglion cells relative to photoreceptor locations introduces spatial offsets between structural and functional measurements, as described in classical anatomical models and subsequent refinements [20,21]. Even when displacement is accounted for, variability related to receptive field size and neural pooling remains. Furthermore, structure–function relationships exhibit non-linear behavior, with ceiling effects in early disease and floor effects in advanced stages, as well as differing dynamic ranges between structural and functional metrics [22]. These physiological constraints likely contribute to the modest correlation strengths observed in anatomically constrained analyses.

Recent advances using deep learning and multimodal imaging have further highlighted the concept of structure–function discordance in glaucoma, demonstrating that functional outcomes cannot always be directly inferred from structural measurements alone. A multimodal Vision Transformer-based model integrating OCT and fundus data showed that prediction accuracy improved after excluding eyes with structural–functional discordance, reinforcing the idea that localized variability represents a fundamental biological and methodological limitation rather than a purely technical artifact [23]. Within this framework, the relatively modest correlations observed in the present point-to-point analysis should be interpreted as reflecting intrinsic heterogeneity of glaucomatous damage rather than insufficient analytical sensitivity.

Several strengths of this study deserve emphasis. Anatomically adjusted overlay incorporating ganglion cell displacement enhanced spatial correspondence between functional and structural data. Layer-specific analysis across multiple intraretinal segmentations enabled detailed characterization of glaucomatous spatial patterns. Strict anatomical inclusion criteria improved geometrical validity and reduced alignment bias.

Nevertheless, important limitations should also be acknowledged. Thickness extraction required manual interaction with the OCT interface, introducing potential operator-dependent variability. The cross-sectional design limits the assessment of longitudinal structure–function evolution. Additionally, the reduced cohort size resulting from strict anatomical inclusion criteria may have limited the detection of subtle localized associations. Furthermore, the single-center design and moderate sample size should be considered when extrapolating these findings to broader populations. Finally, the large number of pointwise correlations evaluated across multiple retinal locations and layers introduces a potential limitation related to multiple comparisons. Although this high-resolution approach was intentionally designed to explore spatial structure–function relationships, it increases the risk of type I error. Therefore, the results should be interpreted with caution, with emphasis on overall spatial patterns rather than isolated statistically significant findings.

Taken together, these findings support a conceptual framework in which anatomically adjusted point-to-point mapping highlights the complex spatial organization of glaucomatous damage while underscoring the physiological limits of localized structure–function coupling.

## 5. Conclusions

Anatomically adjusted point-to-point analysis revealed localized and layer-dependent patterns of macular structure–function coupling in glaucoma, predominantly involving ganglion cell-related layers. Compared with regional analytical approaches, high-resolution point-to-point mapping demonstrated fewer and generally weaker correlations, suggesting that increasing spatial precision may expose intrinsic biological variability rather than strengthen structure–function concordance. These findings support the interpretation of point-to-point analysis as a complementary exploratory strategy that enhances mechanistic understanding of glaucomatous damage while acknowledging the physiological limits of localized structure–function relationships.

## Figures and Tables

**Table 1:** Demographic and Clinical Characteristics of the Study Population.

Demographic Data	Control Group (N = 27)	Glaucoma Group (N = 45)
Age (years)	60.85 ± 17.18	71.07 ± 11.79
Sex (eyes)—Men	8	20
Sex (eyes)—Women	19	25
VA	0.96 ± 0.47	0.81 ± 0.22
IOP (mmHg)	20.07 ± 4.18	17.60 ± 4.62
SE (D)	0.96 ± 1.36	0.13 ± 2.39
C/D ratio	0.50 ± 0.20	0.75 ± 0.19

Data are presented as mean ± standard deviation or number of eyes. VA, visual acuity; IOP, intraocular pressure; SE, spherical equivalent; C/D ratio, cup-to-disk ratio.

## Data Availability

The data sets generated and/or analyzed during this study are available from the corresponding author upon reasonable request.
